# Contrasting Community Composition and Co-Occurrence Relationships of the Active Pico-Sized Haptophytes in the Surface and Subsurface Chlorophyll Maximum Layers of the Arctic Ocean in Summer

**DOI:** 10.3390/microorganisms10020248

**Published:** 2022-01-23

**Authors:** Ping Sun, Yuyu Liao, Ying Wang, Eun-Jin Yang, Nianzhi Jiao, Youngju Lee, Jinyoung Jung, Kyoung-Ho Cho, Jong-Kuk Moon, Dapeng Xu

**Affiliations:** 1 State Key Laboratory of Marine Environmental Science, Xiamen University, Xiamen 361102, China; psun@xmu.edu.cn (P.S.); 22320171150829@xmu.edu.cn (Y.L.); wying@xmu.edu.cn (Y.W.); jiao@xmu.edu.cn (N.J.); 2 Key Laboratory of the Ministry of Education for Coastal and Wetland Ecosystem, College of the Environment and Ecology, Xiamen University, Xiamen 361102, China; 3 Fujian Provincial Key Laboratory for Coastal Ecology and Environmental Studies, Xiamen University, Xiamen 361102, China; 4 Institute of Marine Microbes and Ecospheres, College of Ocean and Earth Sciences, Xiamen University, Xiamen 361102, China; 5 Division of Polar Ocean Science, Korea Polar Research Institute, 26, Songdomirae-ro, Yeonsu-gu, Incheon 21990, Korea; ejyang@kopri.re.kr (E.-J.Y.); yjlee@kopri.re.kr (Y.L.); jinyoungjung@kopri.re.kr (J.J.); kcho@kopri.re.kr (K.-H.C.); jkmoon@kopri.re.kr (J.-K.M.)

**Keywords:** Arctic ecology, community structure, marine biodiversity, protist, microbial eukaryotes, prymnesiophytes

## Abstract

Haptophytes (Hacrobia: Haptophyta), which can perform phototrophic, phagotrophic, or mixotrophic nutritional modes, are critical for element cycling in a variety of aquatic ecosystems. However, their diversity, particularly in the changing Arctic Ocean (AO), remains largely unknown. In the present study, the biodiversity, community composition, and co-occurrence networks of pico-sized haptophytes in the surface water and subsurface chlorophyll maximum (SCM) layer of the AO were explored. Our results found higher alpha diversity estimates in the surface water compared with in the SCM based on high-throughput sequencing of haptophyte specific 18S rRNA. The community composition of the surface water was significantly different from that of the SCM, and water temperature was identified as the primary factor shaping the community compositions. Prymnesiales (mostly *Chrysochromulina*), uncultured Prymnesiophyceae, and *Phaeocystis* dominated the surface water communities, whereas *Phaeocystis* dominated the SCM communities, followed by *Chrysochromulina*, uncultured Prymnesiophyceae, and the remaining taxa. The communities of the surface water and SCM layer developed relatively independent modules in the metacommunity network. Nodes in the surface water were more closely connected to one another than those in the SCM. Network stability analysis revealed that surface water networks were more stable than SCM networks. These findings suggest that SCM communities are more susceptible to environmental fluctuations than those in surface water and that future global changes (e.g., global warming) may profoundly influence the development, persistence, and service of SCM in the AO.

## 1. Introduction

Global estimates indicate that the oceans are responsible for approximately half of the CO_2_ fixed on Earth [1]. As a major source of marine primary production, haptophytes (Hacrobia: Haptophyta) are projected to contribute ca. two-fold more to global oceanic chlorophyll a standing stock in the photic zone of the world oceans compared with either cyanobacteria or diatoms [2,3]. Mixotrophic haptophytes, species that can be both phagotrophy and phototrophy, have been found to be one of the most important picocyanobacterial and bacterial predators in the sea [4,5,6]. 

Certain calcium carbonate scales bearing coccolithophores (e.g., *Emiliania huxleyi*) can absorb atmospheric CO_2_ and sink to the deep ocean via a biological pump, which may have a significant effect on oceanic carbonate production and subsequent global carbon cycling [7]. Some species, e.g., *Phaeocystis pouchetii*, can release toxins that can be harmful to other aquatic organisms, which is more prevalent during bloom-forming conditions [8,9]. Additionally, some species, e.g., *Phaeocystis globose*, are major dimethylsulfoniopropionate (DMSP) producers and hence may influence global sulfur cycling [10]. 

Furthermore, the N_2_-fixing unicellular cyanobacteria (UCYN-A)/haptophyte symbiosis has been found in an increasing variety of marine environments, including the Bering and Chukchi Seas of the Arctic Ocean (AO), and has been proposed to contribute considerably to global nitrogen fixation [11,12,13,14]. The flexible nutritional modes and lifestyles of haptophytes enable them to be one of the most successful microbial eukaryotes, and they are widely dispersed in a variety of marine and freshwater habitats.

As of 2016, ca. 312 species of Haptophyta had been morphologically characterized [15]. Haptophytes are typically considered as single-celled nanophytoplankton (2–20 µm). Only a few pico-sized (≤2–3 µm) species have been described [16]. Due to their small cell size, haptophytes are difficult to study, necessitating observations based on electron microscopy and a high level of taxonomic expertise. Environmental sequencing using universal primers targeting the eukaryotic SSU rRNA gene revealed that sequences affiliated with haptophytes account for only a small portion of all eukaryotic sequences [17]. This contrasts strikingly with the high concentration of 19′-hexanoyloxyfucoxanthin (the accessory photosynthetic pigment found exclusively in haptophytes) in marine waters as detected by high performance liquid chromatography [2,18,19]. The high GC content of haptophyte genomes is proposed to impede amplification processes using universal Eukaryota-specific primer sets, resulting in a low recovery of Haptophyta-specific sequences during environmental surveys. 

With the introduction of haptophyte-specific primers targeting the SSU and LSU rRNA genes, an unexpected diversity was discovered, particularly within the pico-sized fraction. This could represent novel species and lineages at taxonomic levels ranging from genus to class [2,20,21]. Based on plastid SSU rRNA gene data collected from the global ocean, a study recently found two deep-branching plastid lineages, one of which branched close to Prymnesiophyceae and the other branching in a sister position to haptophytes [22].

Marine microorganisms support ocean food webs and drive global biogeochemical cycles by transforming energy and chemical substrates through a multitude of metabolic processes [1]. Recent global warming has had a dramatic and increasing influence on the Arctic Ocean (AO), and the Arctic is warming at nearly double the global average rate [23,24]. Changes in the sea surface temperature have the potential to modify the diversity, composition, and distribution of plankton [25,26]. Alterations to the taxonomic composition of plankton communities can significantly influence critical ecosystem functions, including primary and secondary production, carbon and nutrient cycling, and ultimately ecosystem services [27]. 

Ecological studies on the composition and distribution of nano- and pico-sized eukaryotes have long been a challenge due to their small size and lack of diagnostic features under light microscopy. In the last several decades, the application of sequencing-based techniques, e.g., sequencing on marker genes, such as the SSU rRNA gene, has provided an alternative method for examining the diversity and community composition of these tiny eukaryotic microbes [17,28,29,30]. By sequencing the SSU rRNA rather than the gene, active microbial eukaryotes can be distinguished from dead or dormant cells or extracellular DNA [29,31,32,33,34,35,36,37,38]. Therefore, the RNA-based community is more sensitive to environmental factors than the DNA-based community [39]. Molecular surveys of Arctic microbial eukaryotes have been conducted for more than a decade [40,41,42,43,44,45,46,47]. To the best of our knowledge, no molecular surveys focusing solely on haptophyte assemblages have been conducted, despite the fact that members of this group may play critical roles in the element cycling of AO’s microbial food webs, such as by forming blooms, grazing on other microorganisms, or participating in CO_2_ and nitrogen fixation [13,48].

In the summer of 2016, samples were collected from the surface water and subsurface chlorophyll maximum (SCM) layer of the AO. By applying high-throughput sequencing on the SSU rRNA of pico-sized haptophytes, this study aimed to (1) characterize and compare the community composition and co-occurrence relationships of pico-sized haptophytes in the surface water and SCM and (2) reveal the environmental factors influencing the community of pico-sized haptophytes.

## 2. Materials and Methods

### 2.1. Sample Collection and Measurement of Environmental Parameters

**Figure 1:**
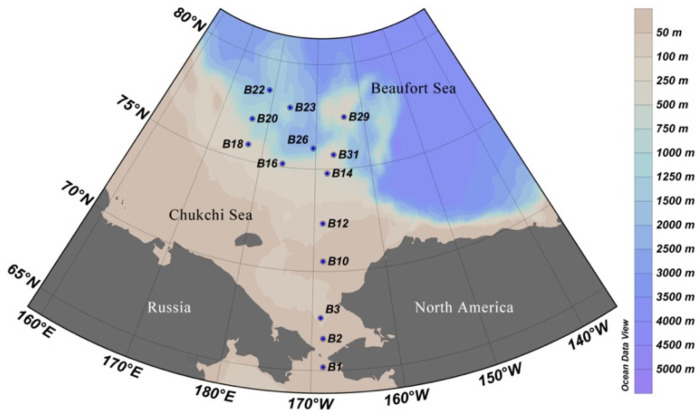
Sampling stations in the Arctic Ocean during the summer cruise of ARA07 conducted in 2016.

Samples were collected on board IBRV ARAON in the summer of 2016 (Expedition ARA07) as detailed in [45,46]. A total of fourteen sites were sampled (Figure 1, Table S1). At each site, seawater from the surface water and subsurface chlorophyll maximum (SCM) layer was sampled using Niskin bottles attached in a circular rosette around the CTD sensors (Sea-Bird SBE 911plus, Sea-Bird Electronics, Bellevue, WA, USA).

The collection and analysis of seawater samples for nutrients (including nitrate + nitrite (NO_2_/NO_3_), phosphate (PO_4_), ammonium (NH_4_), silicate (SiO_4_)), picoplankton (i.e., heterotrophic prokaryotes (HPs), pico-sized pigmented eukaryotes (PPEs)), and sized, fractionated chlorophyll a (i.e., >20 μm, 2–20 μm, and <2 μm fractions), were described in [45].

Water samples for RNA extraction from the surface water and SCM layer were collected as described in [46]. Seawater was prefiltered through a 200 µm mesh (Nitex, Sefar) to remove large metazoans. Five liters of filtrates were then sequentially filtered through 20, 3, and 0.4 μm pore size membrane filters (ISOPORE, Millipore) to collect plankton from the micro- (>20 μm), nano- (3–20 μm), and pico-sized (<3 μm) fractions, respectively. After that, the filters were deep frozen in liquid nitrogen and kept at −80 °C until the RNA extraction. The downstream analysis in this study used only the pico-sized fraction.

### 2.2. RNA Extraction, PCR Amplification, and High-Throughput Sequencing

Environmental RNA was extracted, and the concentration and quality were checked as per [45]. The extracted RNA was immediately reverse transcribed into cDNA using the QuantiTect^®^ Reverse Transcription Kit and gDNA Wipeout Buffer was used to remove the carryover genomic DNA prior to the reverse transcription reaction (Qiagen, Shanghai, China). To amplify the V4 regions (ca. 380 bp) of the 18S rRNA, we used Haptophyta-specific primers (528Flong and PRYM01+7) and the PCR conditions described by [21]. 

Each sample was subjected to five separate PCR reactions to produce sufficient amplicons for sequencing. The Wizard^®^ SV Gel and PCR Clean-Up kit (Promega, Shanghai, China) was then used to excise PCR amplicons from gels. Amplicon libraries were then sequenced in a commercial laboratory using paired-end (2 × 250 bp) sequencing on an Illumina MiSeq sequencer. The reads were submitted to the NCBI SRA with the accession number PRJNA632655.

### 2.3. Data Analysis

Trimmomatic and Flash software were used to perform quality filtering, demultiplexing, and assembly of raw sequences [49,50] with the criteria following [38]. Dereplication of quality-filtered reads was performed on each sample using Usearch 11 [51]. The reads were denoised and clustered into biological zero-radius operational taxonomic units (ZOTUs) using UNOISE3 [52]. 

SINTAX [53] was used to assign taxonomic information to the representative reads of the obtained ZOTUs, and PR2 (Protist Ribosomal Reference database) version 4.11.1 [54], which includes the curated haptophyte reference database, was used as the reference database [55]. The definition of the environmental clades, including Clade_D, Clade_E, Clade_B3, Clade_B4, Clade_HAP2, Clade_HAP3, and Clade_HAP4, followed previous reports [56,57,58,59]. After removing non-Haptophyta ZOTUs, a ZOTU table was generated in USEARCH 11. The ZOTU table was then subsampled for downstream analysis by randomly resampling at the lowest number (35,677) of the reads retrieved for all samples.

QIIME was used to calculate alpha-diversity estimates, such as the ZOTU Richness, Shannon, and Phylogenetic Diversity (PD) [60]. To infer differences between samples, Bray–Curtis distances and Weighted Unifrac distances [61] were calculated to infer sample grouping in R using the ‘vegan’ package, and the results were visualized using a two-dimensional Principal Coordinate Analysis (PCoA). The differences across sample groupings were further tested by ANOSIM within PRIMER 6 [62].

Mantel tests were used to explore the relationships between communities and environmental factors using the ‘vegan’ package in R. The multiple linear regression model (lm function in ‘stats’ package in R [63]) was used in combination with variance decomposition analysis (calc.relimp function in the ‘relaimpo’ package in R [64]) to determine the contributions of the differences in environmental variables in explaining dissimilarities in haptophyte communities.

### 2.4. The Co-Occurrence Network Analyses

To simplify the dataset, ZOTUs with a relative abundance of < 0.01% and found in < 25% of samples were deleted for constructing metacommunity networks. The Spearman correlations between selected ZOTUs were determined using the ‘Hmisc’ [65] and ‘igraph’ packages [66]. Correlations between ZOTUs that were significant (*p*  <  0.01) and robust (ρ  ≥  0.6) were exported as a GML format network file [67,68]. Prior to that, to minimize false positive results, the *p*-values for each network were adjusted with a multiple testing correction using the Benjamini–Hochberg false discovery rate (FDR) control process [69]. 

Gephi v 0.9.2 was used to visualize networks, perform modular analysis, and determine network-level topological properties (i.e., the node, edge, average degree, density, diameter, clustering coefficient, and average path length) [70]. The robustness of the networks was evaluated by simulating a network attack scenario according to [71]. This was accomplished by measuring the natural connectivity of a network when nodes were gradually removed in a predetermined order (according to degree and betweenness) or randomly.

## 3. Results

### 3.1. Environmental Parameters

The environmental parameters of the surface water and SCM layer were reported in [46] (Table S2). The depth of the SCM layer ranged from 15 m at B2 to 62 m at B29. The water temperature of the SCM layer varied between −1.58 and 2.96 °C. The salinity of the SCM layer ranged from 30.7 to 32.4. Except for B2, B3, B10, B12, and B16, the concentration of NH_4_ was below the detection limit in the majority of SCM layer samples. NO_2_/NO_3_ concentrations ranged from 0.7 to 8.22 µM, while PO_4_ concentrations ranged from 0.71 to 2.01 µM. The SiO_2_ concentrations in the SCM layer ranged between 2.55 and 23.74 µM. 

The Chl a concentration in the SCM layer ranged from 0.42 µg L^−1^ at B31 to 6.66 µg L^−1^ at B3, with the 20–200 µm fraction contributing the highest amount at most stations except B26, B29, and B31, where the <2 µm plankton contributed the most. The abundance of heterotrophic prokaryotes (HPs) in the SCM layer ranged between 1.28 × 10^5^ (station B10) to 1.01 × 10^6^ cells mL^−1^ (station B12). The abundance of pico-sized pigmented eukaryotes (PPEs) was about one order of magnitude lower than that of HPs, ranging between 2.12 × 10^3^ and 7.89 cells mL^−1^ (Table S2).

### 3.2. Alpha Diversity and Correlations with Environmental Parameters

Following quality filtering, 2,030,397 reads remained. After removing chimeras, ZOTUs with less than four reads, and non-Haptophyta ZOTUs, 1,845,901 reads were left, ranging from 35,677 to 121,493 reads per sample, which were grouped into 1016 ZOTUs, ranging from 264 to 813 ZOTUs per sample (Table S3). After being rarified at the lowest read count (35,677) across all samples, the number of ZOTUs varied between 198 and 750, with the lowest found in B10 surface water and the highest in B1 surface water. 

**Figure 2:**
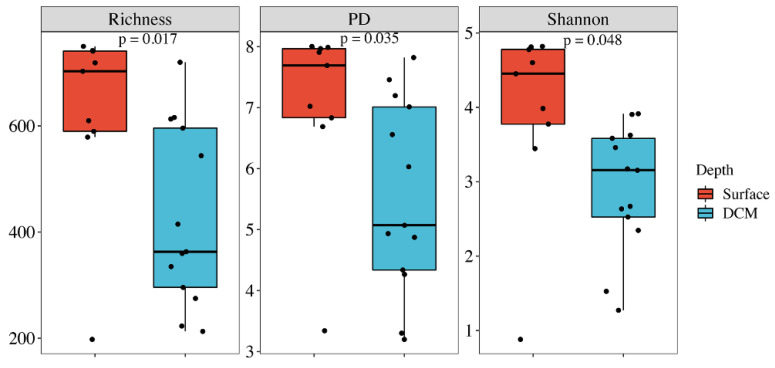
Comparison of the alpha-diversity estimates, including ZOTU Richness, PD, and Shannon, for the pico-sized haptophytes in the surface water and the SCM layer. The line in each box plot indicates the median, and the box delimits the 25th and 75th percentile.

After pooling the samples from the surface water and the SCM layer separately, the alpha diversity estimates for the surface water, including the ZOTU richness, Shannon, and phylogenetic diversity (PD), were significantly higher than those for the SCM layer (Wilcoxon test, *p* < 0.05; Figure 2). All alpha diversity estimates were negatively correlated with salinity (Table S4). Except for ZOTU richness, the PD and Shannon were negatively correlated with the water depth, with the Shannon also being negatively correlated with the NO_2_/NO_3_ concentrations. 

### 3.3. Beta Diversity and Its Driving Factors

**Figure 3:**
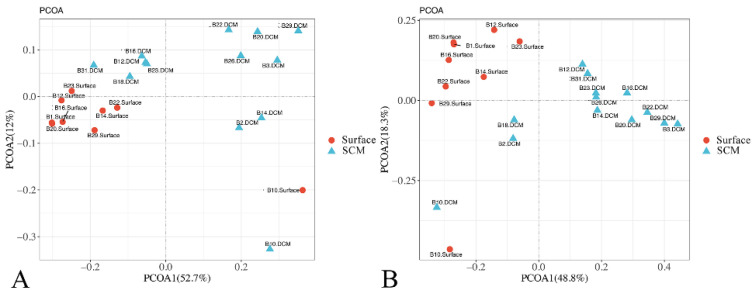
Plots of principal coordinates analysis based on the Bray–Curtis dissimilarities (**A**) and Weighted UniFrac distance matrices (**B**).

Except for station B10, the samples were clustered into two groups, i.e., the surface water group and the SCM layer group, based on their Bray–Curtis dissimilarity (Figure 3A). This grouping pattern was also supported by the principal component analysis (PCoA) of community taxonomic relatedness quantified by the Weighted Unifrac metric (Figure 3B). Statistical analysis revealed a substantial difference in the composition of the surface water and SCM layer samples (ANOSIM, R = 0.343, *p* = 0.004 for the Bray–Curtis dissimilarity and R = 0.540, *p* = 0.001 for the Weighted Unifrac distance).

The Mantel test was used to determine the influence of environmental parameters on haptophyte communities. Temperature was identified to be the primary driving factor (*p* = 0.002, R^2^ = 0.448), followed by Chl a (2–20 µm) (*p* = 0.002, R^2^ = 0.162) (Table 1). Additionally, the multiple linear regression analysis showed that water temperature was the most important driving factor, accounting for ca. 12.9% of the community variance, followed by other measured environmental parameters, which completely explained ca. 25.8% of the community variance (Table 2).

### 3.4. Community Composition

**Figure 4:**
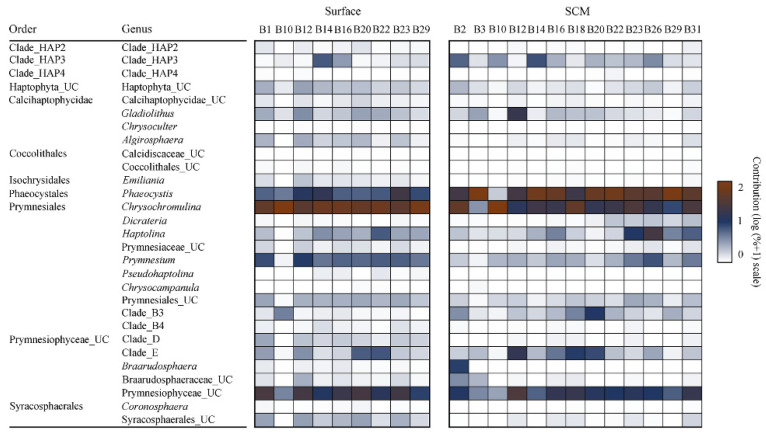
Heatmap showing the compositions of major pico-sized haptophyte communities in the surface water and SCM layer.

To infer the community composition of pico-sized haptophytes, all representative reads for each ZOTU were searched against the PR2 database, and their identities at the lowest taxonomic levels were determined. The haptophyte-specific reads were categorized into 29 phylogenetic taxa, with 13 at the genus level and 7 at the environmental clade level, including Clade_HAP2-4, Clade_B3-B4, and Clade_D-E. The rest were assigned as unclassified haptophytes at the class or order level (Figure 4). Most of the sites had a diverse haptophyte community. 

The surface community was dominated by *Chrysochromulina*, followed by Prymnesiophyceae_UC, *Phaeocystis*, *Prymnesium*, and other taxa. In the SCM layer, *Phaeocystis* surpassed *Chrysochromulina* as the most dominant genus, followed by Prymnesiophyceae_UC and other taxa. Environmental clades, including Clade_HAP3, Clade_B3, and Clade_E, contributed more to the SCM layer than to the surface water communities, while Clade_D was more prevalent in the surface water community. The coccoliths bearing Calcidiscaceae_UC, Coccolithales_UC, and *Emiliania* were virtually missing from both the surface water and SCM layer communities. Within Prymnesiales, the uncultured *Chrysochromulina* (*Chrysochromulina*_UC) predominated in both the surface water and the SCM layer communities (Figure S1A). The uncultured *Prymnesium* (*Prymnesium*_UC) was the second most-abundant genus in surface water among the Prymnesiales, with the others contributing only marginally to the community. In the surface water of the B10 station (B10.Surface), the environmental clades Clade_B3-B4-B5 were the second most abundant taxa. In the SCM layer, *Chrysochromulina leadbeateri* was the second most abundant species within Prymnesiales in some samples (e.g., B23.DCM, B2.DCM, and B10.DCM) but was replaced by the uncultured *Haptolina* (*Haptolina*_UC) in certain samples (e.g., B29.DCM, B31.DCM, and B26.DCM) to be the second most abundant genus (Figure S1A). Within Phaeocystales, surface water samples were dominated by the uncultured *Phaeocystis* (*Phaeocystis*_UC) in most samples, whereas *Phaeocystis pouchetii* was also prominent in some samples (Figure S1B). The composition of Phaeocystales in the SCM layer was strikingly different from that in the surface water samples with *Phaeocystis pouchetii* being the most abundant species in most samples (Figure S1B). 

In terms of the ZOTU richness, the composition of pico-haptophytes varied little between samples. Prymnesiales and Prymnesiophyceae_UC both contributed nearly equally to the community, which in total accounted for >80% of the ZOTUs detected in each sample. The remaining taxa made only a marginal contribution to the community (Figure S2).

**Figure 5:**
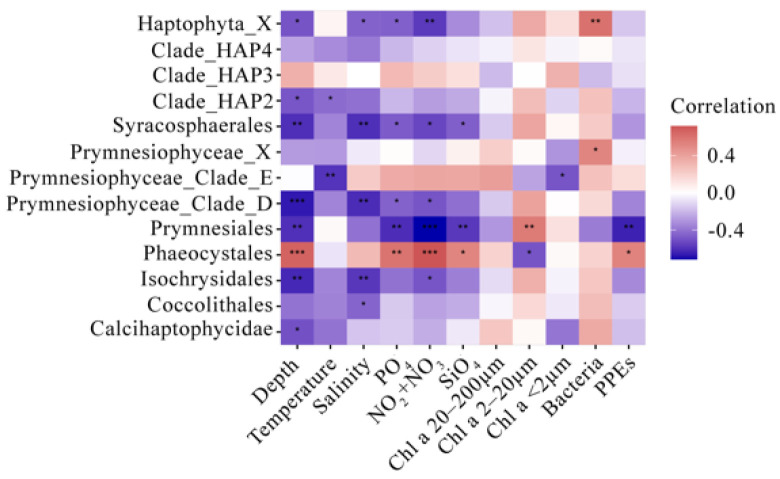
Heatmap showing Spearman’s correlations between the relative abundance of major pico-sized haptophyte taxa and environmental parameters. The correlation coefficient values are indicated according to the color bar.

Spearman’s correlation analysis was conducted to explore the possible influence of environmental variables on the relative sequence abundance of key taxa (Figure 5). Water depth was typically negatively correlated with several taxa, including Haptophyta_X, Clade_HAP2, Syracosphaerales, Clade_D, Prymnesiales, Isochrysidales, and Calcihaptophycidae, but was positively correlated with Phaeocystales. The temperature negatively affected only Clade_HAP2 and Clade_E. Except for Phaeocystales, which was usually positively correlated with the above factors, most groups responded negatively to depth, temperature, salinity, and nutrients. Certain taxa, including Clade_HAP4 and Clade_HAP3, were found to have no significant correlations with any of the factors measured.

### 3.5. Co-Occurrence Networks

**Figure 6:**
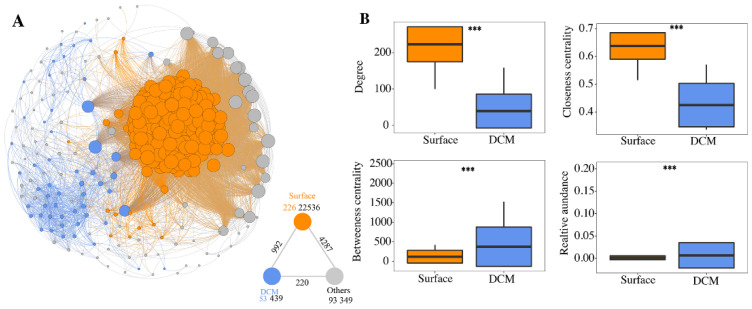
(**A**) Co-occurrence network of pico-sized haptophytes. Each node is proportionate in size to the degree of the ZOTUs. The triangle graph in the lower right corner summarizes the node-edge statistics. The color number indicates the number of nodes in the associated category. Dark gray numbers indicate the number of inner-connections, whereas the numbers adjacent to the edge connections represent cross-group interactions. (**B**) Comparison of the node-level network topological properties between surface-water- and SCM-enriched ZOTUs. *** donates *p* < 0.001.

Based on correlation relationships, a metacommunity co-occurrence network was constructed, capturing 23,324 associations among 372 haptophyte ZOTUs (Figure 6A). A total of 226 and 53 significantly enriched ZOTUs in surface water and the SCM layer were identified, respectively. Surface-water- and SCM-enriched ZOTUs formed distinct modules, with surface-water-enriched ZOTUs exhibiting much closer interconnections than SCM-enriched ZOTUs. Additionally, we examined the node-level topological parameters of different groups of ZOTUs (Figure 6B). The values of topological parameters, including the degree and closeness centrality, were significantly higher (*p* < 0.01) in surface-water-enriched ZOTUs compared to SCM-enriched ZOTUs. 

Subnetworks were generated for surface-water- and SCM-enriched communities, and a set of network-level topological parameters were calculated (Table S5). The average degree, clustering coefficient, and graph density of the surface water subnetwork were significantly higher than those of the SCM subnetwork, implying that surface-water-enriched ZOTUs were more interconnected. The average path length and diameter were lower in the surface water subnetwork, indicating that surface-water-enriched communities are more closely related.

Additionally, we simulated a network attack scenario to examine the stability of surface water and SCM layer networks. With the removal of critical nodes with high betweenness and degree, the SCM layer network lost connectedness more rapidly than the surface water network (Figure S3A). The random attacking scenario revealed a similar pattern (Figure S3B): the natural connectedness of the surface water network was constantly greater than that of the SCM layer network under increasing random node loss, thus, suggesting greater robustness of the surface water network.

## 4. Discussion

The SCM layer is typically characterized as the region beneath the surface water that has a maximum of chlorophyll fluorescence at depth, which is a common feature of most aquatic ecosystems, particularly those with strong thermal stratification [72]. The depth of the SCM was traditionally viewed as a trade-off between available light and nutrients for phytoplankton growth [72]. The persistence of SCM may be highly susceptible to changes in the water column’s physical properties. One of the most noticeable hydrological characteristics in the western AO in summer is the formation and persistence of the SCM layer, which is responsible for most of the primary production [73]. Any perturbance of this sensitive light-nutrient balance is expected to change the community structure [74], hence, affecting essential ecosystem functions, such as the primary and secondary production as well as element cycling [27]. Research reported that microeukaryotic phytoplankton were the predominant primary producers in the western AO [75,76]. Thus, understanding the community composition, co-occurrence relationships, and environmental drivers of microeukaryotic assemblages, particularly haptophytes, is critical for appreciating their functions in a changing AO.

### 4.1. Beta Diversity, Taxonomic Composition, and Environmental Driving Factors

The striking difference in temperature, salinity, nutrients, and light between the surface water and SCM layer has resulted in distinct community compositions of microbial eukaryotes in diverse marine environments, including the AO [41,46,77,78]. In the Southeast Pacific Ocean, Red Sea, Mediterranean Sea, Norwegian coast, and the central Pacific Ocean, previous studies have reported contrasting haptophyte communities in the surface water and SCM layer [20,55,59,79,80]. Separation of surface water and SCM-layer haptophyte communities was also observed in this study, as determined by PCoA plotting of both the Bray–Curtis dissimilarities and Weighted Unifrac distance, which is consistent with earlier reports (Figure 3). 

The mantel test identified temperature as the most influential factor shaping the haptophyte communities in the AO, which was corroborated by the multivariate multiple linear regression analysis, which showed that temperature explained 12.9% of the observed variations. The influence of temperature on community structure may be partially explained by how species’ performance (fitness) responds to temperature variations, i.e., the species’ thermal tolerance curves [81].

Prymnesiales were the most abundant taxon in most of the surface water samples and the second most abundant taxon in the majority of SCM samples, which is consistent with previous reports (Figure 4). *Chrysochromulina* and *Prymnesium* were the two most abundant genera in the Prymnesiales encountered in this study. These two genera were previously recognized for their high diversity and widespread distribution [15]. Some species of these two genera, e.g., *Chrysochromulina hirta*, *Chrysochromulina ericina*, *Prymnesium patelliferum*, and *Prymnesium parvum*, have been reported to be capable of hunting for prey or ingesting organic particles via phagocytosis using the haptonema, allowing them to adapt to the low light/nutrient environments [82,83,84,85,86]. 

Indeed, throughout our study, the nutrients PO_4_, NO_2_/NO_3_, and SiO_4_ were much lower in the surface water than in the SCM layer (Table S2). The ability of Prymnesiales, especially species of the genera *Chrysochromulina* and *Prymnesium*, to perform both phototrophy and phagotrophy may enable them to outcompete species that can only perform phototrophy or phagotrophy and may account for their dominance in the low nutrient surface water of the AO. *Phaeocystis* within the Order Phaeocystales replaces Prymnesiales as the highest contributor to the SCM layer in most of the samples. Most of the *Phaeocystis* ZOTUs recovered in the SCM layer were classified as *Phaeocystis pouchetii* (Figure S1). *P. pouchetii* is a phytoplankton species that lives in cold waters in the northern hemisphere. 

In the AO, *P. pouchetii* can form spring blooms, contributing to primary production, the sedimentation of organic carbon, and the food supply for zooplankton [87,88,89]. Although this species has not been observed to undertake phagotrophy, the comparatively rich nutrients and ample light in the SCM layer enabled *P. pouchetii* to proliferate rapidly, displacing *Chrysochromulina* and *Prymnesium* as the dominating species in the SCM layer. Indeed, previous studies suggested that haptophytes, particularly the non-calcifying taxa, including *Chrysochromulina* and *Prymnesium*, were primarily K-strategists capable of mixotrophy and well-adapted to conditions of intermediate or low nutrition availability and turbulence [80,90]. 

On the contrary, bloom-forming species, such as *P. pouchetii*, that can grow fast and achieve high abundances under optimum conditions (e.g., nutrients and light), are most likely R-strategists [90]. Indeed, in the Spearman’s correlation analysis between environmental variables and the relative sequence abundance of major taxonomic groups, Prymnesiales and Phaeocystales responded oppositely, with the former being negatively correlated with water depth and nutrients and the latter being positively correlated with these environmental factors (Figure 5). The distinct niche preferences of Prymnesiales and Phaeocystales, as well as the contrasting environmental conditions between the surface water and SCM layer, may explain the different distribution patterns of these two haptophyte taxa observed in the present study.

### 4.2. Co-Occurrence Networks

In recent decades, co-occurrence networks, which can reveal information on associations among microbial communities and the stability of communities, have been increasingly used to infer potential interactions of microbial assemblages in a variety of terrestrial and aquatic environments [37,46,91,92,93,94,95,96]. In this study, we constructed co-occurrence networks based on high-throughput sequencing on 18S rRNA of pico-sized haptophytes. By using the 18S rRNA instead of the gene, we can avoid interference from dead, dormant cells, and extracellular free DNA, thus, allowing us to analyze only the “active” members of the community [29,31,32,33].

Similar to the community structure, the co-occurrence networks displayed different patterns with distinct network properties and stability between the surface water and SCM layer (Figure 6). A total of 226 and 53 highly enriched ZOTUs were detected in the surface water and SCM layer, respectively, which is consistent with the fact that higher surface water communities had higher alpha diversity estimates than the SCM layer communities. The surface-water- and SCM layer-enriched ZOTUs formed two largely independent modules in the co-occurrence network (Figure 6). 

According to our findings, the surface water network had a higher average degree, clustering coefficient, and graph density than the SCM network, according to our findings. Additionally, the degree and closeness centrality of surface-water-enriched ZOTUs were higher than those of SCM-enriched ZOTUs. All the aforementioned findings revealed that the pico-sized haptophytes within the surface water and SCM layer were more closely connected than those between the two groups. 

This is congruent with the sample grouping determined by the Bray–Curtis dissimilarity and Weighted Unifrac distance analyses, which showed that the surface water and SCM layer communities were well separated. The SCM is persistent in the western AO during the summer [73]. The strongly salinity stratified seawater can result in distinct physical and chemical properties between the surface water and the SCM layer, which may select microbial eukaryotes that adapt to the surrounding seawater and the assembly processes of microbial eukaryotic communities resulting in the separation of the surface water and SCM layer microbial eukaryotic communities [97].

Our results showed that, with the removal of both critical nodes and the random nodes, the surface water networks lost connections more slowly than the SCM networks. As a result, the surface water networks were likely more robust, i.e., stable, compared with the SCM networks. It has been suggested that high diversity can facilitate the co-occurrence of microbial communities [98], and ecosystems with higher levels of biodiversity are more stable [99,100]. 

Indeed, we found significantly higher alpha diversity estimates, including the ZOTU richness, Shannon, and PD, in the surface water than in the SCM layer. Additionally, a previous study revealed that communities with low diversity may be more susceptible to fast change than those with higher diversity [101]. Thus, communities in the SCM layer, which have lower diversity and more unstable co-occurrence network relationships among species compared with surface water communities, are likely to be more sensitive to environmental changes, e.g., inflow from the melting sea ice and river discharge that add freshwater to the AO. Changes in the microbial assemblages inhabiting the SCM are anticipated to influence the key ecological processes of the SCM, e.g., the primary production. Our findings corroborate a previous report indicating that increased terrigenous input may influence the development of SCM and result in a more heterotroph dominated microbial eukaryotic community, thereby, resulting in decreased primary production [97].

## 5. Conclusions

In the present study, high-throughput sequencing of haptophyte specific 18S rRNA was used to investigate the biodiversity, community composition, and co-occurrence relationships of pico-sized haptophytes in the AO’s surface water and SCM layer. Our data found that the surface water had higher alpha diversity estimates compared with the SCM layer. The surface water and SCM layer were found to harbor distinct communities with water temperature being the primary driving factor. 

The surface water and SCM layer communities formed relatively independent modules in the metacommunity network with the surface water networks being more stable than the SCM networks. These findings imply that SCM communities are more susceptible to environmental fluctuations compared with surface water, and that future global changes (e.g., global warming) may have a profound impact on the development, persistence, and service of SCM in the AO.

## Figures and Tables

**Table 1:** Mantel test comparison between the haptophyte community variability (measured as the Bray–Curtis dissimilarity) and environmental parameters.

Environmental Parameters	R^2^	*p*
Geographic distance	0.006	0.415
Depth	0.180	0.055
Temperature	**0.448**	**0.002**
Salinity	0.098	0.149
PO_4_	0.096	0.194
NO_2_ + NO_3_	0.040	0.313
SiO_2_	0.072	0.216
Chl a (>20 µm)	0.086	0.111
Chl a (2–20 µm)	**0.162**	**0.046**
Chl a (<2 µm)	0.151	0.081
HPs, abundance	0.029	0.365
PPEs, abundance	0.108	0.167

HPs, heterotrophic prokaryotes; and PPEs, pigmented picoeukaryotes. Numbers in bold indicate statistically significant results.

**Table 2:** The results of multivariate multiple linear regression (MLR) performed between environmental variables (temperature, Chl a (<2 μm), Chl a (2–20 μm), Chl a (>20 μm), PPEs, heterotrophic bacteria, salinity, and geographic distance)) and community dissimilarities. Explanatory variables were normalized, and the Euclidean distance was calculated.

Variable	Cumulative %
Temperature	12.9
Chl a (<2 μm)	4.3
Chl a (2–20 μm)	2.6
PPEs	1.5
Chl a (>20 μm)	1.4
Heterotrophic bacteria	1.3
Salinity	1.3
Geographic distance	0.5

## Data Availability

Reads generated from high-throughput sequencing were deposited in the NCBI SRA under the accession number PRJNA632655.

## Supplementary Materials

The following supporting information can be downloaded at: https://www.mdpi.com/article/10.3390/microorganisms10020248/s1, Figure S1: Taxonomic compositions of Prymnesiales and Phaeocystales. Figure S2: The proportions of ZOTUs. Figure S3: Network stability was measured for surface water and SCM layer pico-haptophyte communities using a network at-tacking scenario in which nodes were gradually removed in a predetermined order or randomly. Table S1: Sample Information: SCM—subsurface chlorophyll maximum layer. Table S2: Environmental parameters at the sampling stations. Table S3: Alpha diversity estimates of the samples collected. *Reads before randomly subsampling of 35,677 sequences without replacement. Table S4: Spearman correlations between environmental factors and alpha diversity estimates of pico-sized haptophytes revealed by the SSU rRNA. Table S5: The topological features of co-occurrence networks for pico-sized haptophytes in surface water and SCM layer.
