# Supplementary material for: Contrasting Community Composition and Co-Occurrence Relationships of the Active Pico-Sized Haptophytes in the Surface and Subsurface Chlorophyll Maximum Layers of the Arctic Ocean in Summer
Source: Microorganisms. 2022 Jan 23;10(2):248. doi: 10.3390/microorganisms10020248 (PMC8877492; doi:10.3390/microorganisms10020248)
Supplement: Supplementary file 1 [file microorganisms-10-00248-s001.zip › microorganisms-1540055-supplementary.pdf]

microorganisms

MDPI

Supplementary Material

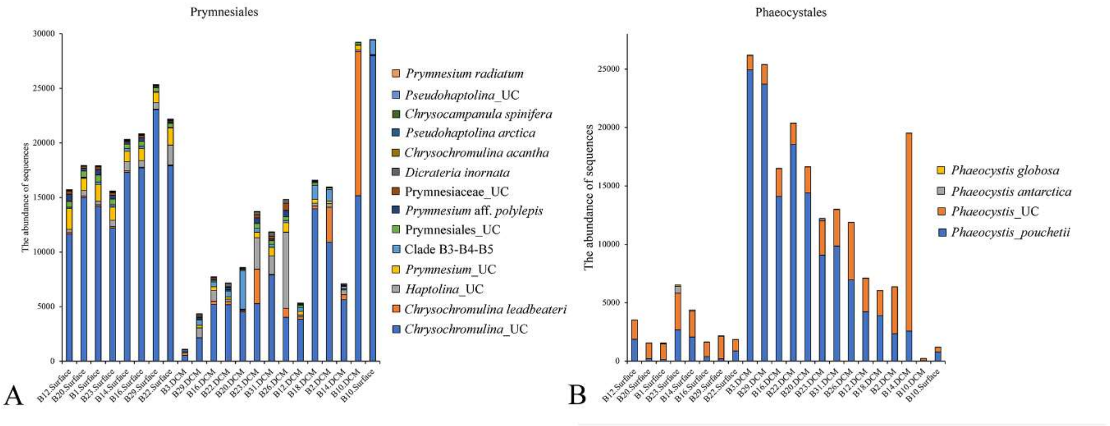

Figure S1. Taxonomic compositions of Prymnesiales (A) and Phaeocystales (B).

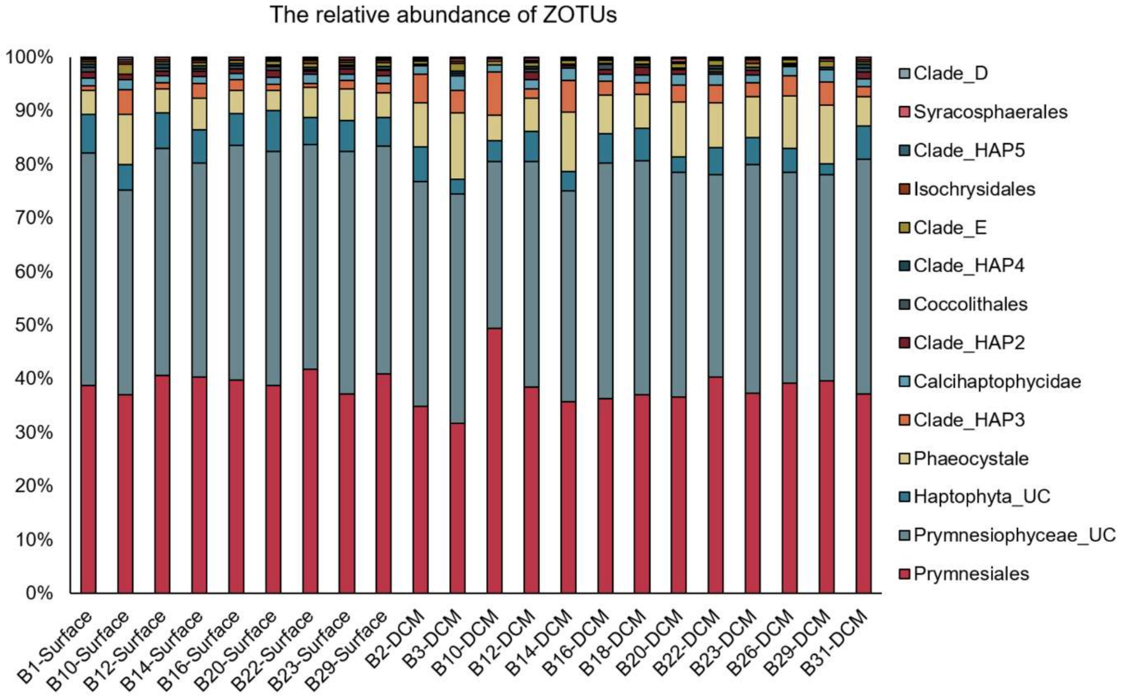

Figure S2. The proportions of ZOTUs.

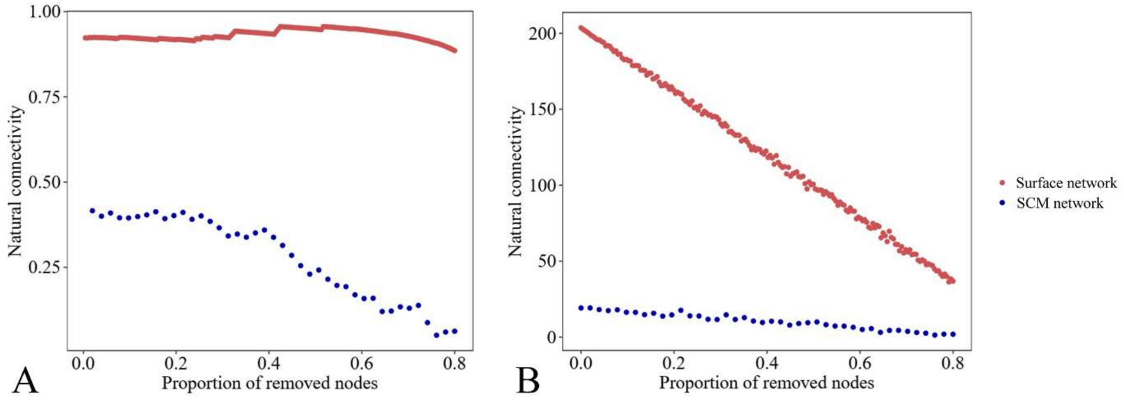

Figure S3. Network stability was measured for surface water and SCM layer pico-haptophyte communities using a network attacking scenario in which nodes were gradually removed in a predetermined order (A) or randomly (B).

Table S1. Sample Information: SCM—subsurface chlorophyll maximum layer.

Sample ID	Station	Latitude	Longitude	Depth (m)
B1-Surface	B1	65.1735	-168.6907	0
B2-SCM	B2	66.6299	-168.6875	15
B3-SCM	B3	67.6700	-168.9600	25
B10-Surface	B10	70.5000	-168.6667	0
B10-SCM				25
B12-Surface	B12	72.3600	-168.6667	0
B12-SCM				35
B14-Surface	B14	74.8006	-167.8983	0
B14-SCM				32
B16-Surface	B16	75.1500	-176.0000	0
B16-SCM				50
B18-SCM	B18	75.7669	177.0685	37
B20-Surface	B20	77.0000	176.5700	0
B20-SCM				27
B22-Surface	B22	78.5218	178.8452	0
B22-SCM				35
B23-Surface	B23	77.8664	-175.9087	0
B23-SCM				44
B26-SCM	B26	76.0000	-170.5000	55
B29-Surface	B29	77.4723	-164.1180	0
B29-SCM				62
B31-SCM	B31	75.6912	-166.6382	44

Table S2. Environmental parameters at the sampling stations.

			Nutrients (μM)				Chl *a* (μg/L)					
**Sample ID**	**T (°C)**	**S**	**PO_4**	**NO_2+NO_3**	**NH_4**	SiO_4	Total	> 20 μm	2-20 μm	< 2 μm	Bacteria (cells/mL)	PPEs (cells/mL)
B1-Surface	9.40	31.0	0.25	0.00	0.00	3.08	0.50	0.19	0.22	0.09	1.28E+06	1.46E+04
B10-Surface	6.51	31.2	0.24	0.00	0.00	1.00	0.10	0.22	0.15	0.13	1.78E+05	3.39E+03
B12-Surface	-0.74	28.5	0.54	0.00	0.00	9.48	0.28	0.36	0.05	0.09	8.16E+05	3.28E+03

B14-Surface	-1.09	27.5	0.55	0.00	0.00	1.52	0.09	0.10	0.22	0.18	2.47E+05	3.32E+03
B16-Surface	-1.14	28.2	0.63	0.00	0.00	0.49	0.07	0.13	0.21	0.15	2.03E+05	1.79E+03
B20-Surface	-1.51	30.0	0.61	0.00	0.00	0.00	0.40	0.09	0.33	0.07	2.24E+05	4.81E+03
B22-Surface	-1.45	29.2	0.46	0.00	0.00	0.66	0.08	0.14	0.23	0.13	2.20E+05	1.40E+03
B23-Surface	-1.34	28.4	0.61	0.00	0.00	1.38	0.06	0.11	0.22	0.17	2.22E+05	1.10E+03
B29-Surface	-1.25	27.4	0.55	0.03	0.00	2.04	0.05	0.07	0.24	0.19	1.69E+05	1.26E+03
B2-SCM	2.96	31.8	0.87	1.05	1.54	12.38	0.52	0.24	0.15	0.11	8.55E+05	4.07E+03
B3-SCM	2.06	32.2	0.84	1.71	0.21	10.88	6.66	0.45	0.04	0.01	4.62E+05	7.89E+03
B10-SCM	2.41	32.1	0.84	0.70	0.47	9.05	0.68	0.34	0.10	0.05	1.28E+05	2.12E+03
B12-SCM	-1.17	32.4	2.01	8.22	4.03	23.74	2.14	0.42	0.07	0.01	1.01E+06	4.66E+03
B14-SCM	-0.88	30.7	1.00	3.18	0.00	7.65	0.43	0.07	0.18	0.25	3.42E+05	4.92E+03
B16-SCM	-1.45	31.7	1.12	5.75	0.17	11.04	1.89	0.37	0.08	0.05	1.86E+05	5.03E+03
B18-SCM	-1.48	31.7	1.29	3.97	0.00	9.65	1.07	0.40	0.08	0.01	2.17E+05	4.36E+03
B20-SCM	-1.58	31.7	1.08	4.84	0.00	14.72	0.74	0.36	0.08	0.06	1.49E+05	3.56E+03
B22-SCM	-0.80	31.5	0.83	2.96	0.00	8.39	1.24	0.36	0.09	0.06	2.35E+05	5.72E+03
B23-SCM	-0.21	31.1	0.71	1.01	0.00	2.55	1.07	0.29	0.09	0.12	4.03E+05	7.46E+03
B26-SCM	-0.35	31.0	0.74	1.01	0.00	4.00	0.66	0.14	0.12	0.24	3.35E+05	6.90E+03
B29-SCM	0.17	30.9	0.83	1.37	0.00	5.91	0.43	0.05	0.12	0.33	1.89E+05	7.93E+03
B31-SCM	-1.16	30.9	1.06	2.98	0.00	7.31	0.42	0.16	0.17	0.17	2.62E+05	4.12E+03

Table S3. Alpha diversity estimates of the samples collected. *Reads before randomly subsampling of 35,677 sequences without replacement.

Sample	Reads*	ZOTU*	ZOTUs	Coverage	Shannon	PD
B1-Surface	46710	785	750	0.9967	4.82	8.00
B10-Surface	77767	264	198	0.9982	2.67	3.34
B12-Surface	64681	788	741	0.9977	0.88	7.97
B14-Surface	35677	610	610	0.9962	3.46	7.02
B16-Surface	65324	782	719	0.9968	4.81	7.91
B20-Surface	60491	755	703	0.9967	2.52	7.69
B22-Surface	73268	682	590	0.9962	3.99	6.84
B23-Surface	49330	788	742	0.9962	3.16	7.99
B29-Surface	65416	648	579	0.9966	4.45	6.69
B2-SCM	106031	500	363	0.9964	3.91	4.88
B3-SCM	96255	402	275	0.9970	3.62	4.34
B10-SCM	121493	319	213	0.9969	2.64	3.20
B12-SCM	58364	632	544	0.9970	4.6	6.56
B14-SCM	111701	419	296	0.9960	2.35	4.27
B16-SCM	102869	711	596	0.9957	3.78	7.01
B18-SCM	102190	753	616	0.9955	3.58	7.46
B20-SCM	104469	480	335	0.9972	4.78	4.93
B22-SCM	113514	579	415	0.9966	3.17	6.03
B23-SCM	114902	765	613	0.9978	1.53	7.20
B26-SCM	103266	496	360	0.9967	3.45	5.07
B29-SCM	90575	316	223	0.9970	1.27	3.30
B31-SCM	81608	813	720	0.9962	3.91	7.82

Table S4. Spearman correlations between environmental factors and alpha diversity estimates of pico-sized haptophytes revealed by the SSU rRNA.

	**ZOTU Richness**		PD		Shannon	
	**r**	***p***	r	*p*	r	*p*
Latitude	0.141	0.530	0.148	0.512	-0.013	0.954
Longitude	-0.155	0.490	-0.176	0.432	-0.128	0.570
Depth	-0.403	0.063	**-0.433**	**0.044**	**-0.505**	**0.017**
Temperature	-0.331	0.132	-0.343	0.118	-0.249	0.264
Salinity	**-0.480**	**0.024**	**-0.485**	**0.022**	**-0.488**	**0.021**
PO_4	-0.243	0.276	-0.287	0.196	-0.321	0.146
NO_2+NO_3	-0.341	0.120	-0.370	0.090	**-0.498**	**0.018**
SiO_4	-0.298	0.179	-0.334	0.129	-0.327	0.138
Chl *a*, > 20 μm	-0.076	0.737	-0.049	0.830	-0.166	0.461
Chl *a*, 2-20 μm	0.289	0.192	0.271	0.223	0.390	0.073
Chl *a*, < 2 μm	-0.005	0.984	-0.022	0.922	0.029	0.899
Heterotrophic bacteria	0.322	0.143	0.292	0.187	0.337	0.125
PPEs	-0.164	0.465	-0.170	0.450	-0.304	0.168

Table S5. The topological features of co-occurrence networks for pico-sized haptophytes in surface water and SCM layer.

	**Node**	**Edge**	**Average degree**	**Clustering coefficient**	**Average path length**	**Diameter**	**Graph density**
Whole	372	28823	154.962	0.807	1.93	6	0.418
Surface	226	22536	199.434	0.964	1.114	3	0.886
DCM	51	439	17.216	0.784	1.991	4	0.344
